# Exploring the Needs of Young People Born With Cleft Lip and/or Palate Approaching End of Routine Care, in the UK

**DOI:** 10.1177/10556656241312494

**Published:** 2025-01-17

**Authors:** Julie Davies, Elaine Davidson, Sam Harding, Yvonne Wren, Lucy Southby

**Affiliations:** 1 152359 Bristol Dental School, University of Bristol, Bristol, UK; 2 North West North Wales and Isle of Man Cleft Network, Manchester, UK; 3 Cleft-NET-East, 2153 Cambridge University Hospitals NHS Foundation Trust, Cambridge, UK; 4 Bristol Speech and Language Therapy Research Unit, 1982 North Bristol NHS Trust, Bristol, UK; 5 Cardiff School of Sport and Health Sciences, 11352 Cardiff Metropolitan University, Wales, Cardiff, UK

**Keywords:** cleft lip and palate, nominal group technique, reflexive thematic analysis, transition to adult care, approaching end of routine care

## Abstract

**Objective:**

The aim of this study was to understand the needs of young people, aged 16 to 20 years, born with cleft lip and/or palate (CL/P) approaching end of routine care, in the United Kingdom (UK).

**Design:**

Nominal group technique was used during 2 online focus groups to identify priorities for cleft care at transition to adult care. Focus group discussions were recorded, transcribed, and analyzed using reflexive thematic analysis.

**Participants:**

Ten young people born with CL/P, 8 aged 18 to 20 years and 2 aged 16 to 17 years were recruited via the UK-based cleft charity, the Cleft Lip and Palate Association (CLAPA).

**Results:**

Three themes were identified from the analysis: Theme 1—Psychological well-being is not a constant. Theme 2—Asking and listening—the cleft clinic appointment. Theme 3—Approaching end of routine care and re-accessing cleft services.

**Conclusions:**

Findings suggest that young people have ongoing needs in older adolescence. Psychological well-being, taking control of their own treatment and re-accessing the cleft team were highlighted as particular issues. Whilst further research with a larger and more representative sample is needed, these results support the need for continued access to and provision from the cleft team at this age.

## Introduction

Orofacial clefts are the most common congenital anomaly affecting the face, with over one thousand babies every year being born in the United Kingdom (UK) with a cleft of the lip or palate or both (CL/P).^1^ It is a lifelong condition and some individuals born with CL/P may continue to receive treatment through childhood, adolescence, and into adulthood, with on average, 8.6 surgical procedures before age 21.^2^ Treatments are often concentrated in the early years of life with important interim outcomes, for example, speech, dental health, hearing, facial growth, and wellbeing, commonly measured in childhood.^3–8^ What happens for young people (YP) as they approach end of routine care, as well as what they need from cleft care services, is less well understood.^9^ For the purposes of this study, the term YP includes individuals in older adolescence and early adulthood from age 16 to 20 years.

In the UK, the cleft care pathway extends from pre-birth to adult life and involves a multidisciplinary team (MDT) approach, with clinicians from the specialties of Nursing, Surgery, Speech and Language Therapy, Psychology, Dentistry, Orthodontics and Audiology.^10^ Specialist cleft services in the UK continue to be available into adulthood either to continue ongoing care from childhood/adolescence or to be re-accessed if new concerns arise later in adulthood. However, when routine follow-up comes to an end, YP born with CL/P must be proactive in seeking support rather than responsive to invitations to appointments.

The period between ages 16 to 20 years is important because in the UK, YP can formally consent for their own healthcare treatment from the age of 16 years which, for some, coincides with discharge from routine cleft care follow-up. Generally, routine care ends before the child is aged 20, or soon after, but this varies depending on cleft subtype and individual needs for treatment from any member of the MDT. Discharge should not be considered a cutting of care, but an ending of the current phase with an invitation to return later, if wanted. In addition, more general milestones occur around this age, including the end of formal education, moving into employment, higher education and/or independent living which may or may not be in the same geographical location as the previous years. This transitional period is mirrored in many international contexts, albeit with variation depending on exact age boundaries and national health and education systems.^11^ Understanding longer-term outcomes and ongoing needs of individuals as they approach adulthood is important to inform services.^11^ Concerns regarding appearance, speech, teeth, hearing, and well-being may all persist, or new concerns may arise in adolescence and adulthood for YP with CL/P.^12–15^ Several studies, covering a wide age range of participants, have explored the experiences and quality of life of YP living with cleft conditions.^14,16–19^ Some of this work has been used to support the development of patient reported outcome measures,^14^ through assessing Quality of Life measures (physical, psychological, and social health parameters). As with other long-term or life-long conditions, YP born with CL/P become more involved in treatment decisions related to their cleft condition, eventually becoming independent in the management of their care.

Previous studies looking at the end of routine CL/P care have reported : feeling unprepared for the transition; a lack of information about how to seek support that might have helped with making decisions about further treatment; and ways to balance the challenges of cleft care with other aspects of their life.^20,21^ A recent study comparing the experiences of individuals aged 16 to 25 years, born with CL/P, with the National Institute for Clinical Excellence (NICE) guidance for health services in managing and supporting the transition from children to adult services, found that although some aspects were being implemented, many recommendations were often not met,^22^ perhaps reflecting challenges faced by services such as limited resources or funding. Seeking the views of YP on what they consider important to be included in cleft care appointments at this age, and what their key needs are, could inform the care that specialist cleft services provide and contribute to decisions on what services or support may be needed or developed.

The aim of this research was to identify factors that YP born with CL/P, aged 16 to 20 years and living in the UK, consider to be important in relation to their cleft care. This was the first stage of a bigger research program which will look at outcomes for YP at the end of routine care. This first step was conducted to understand patients’ views so that it could feed into the larger research program which will use a mixed methods approach to understanding outcomes of YP born with a cleft condition.

## Method

A qualitative study involving focus groups and using nominal group technique (NGT) combined with reflexive thematic analysis (RTA) was employed to address the aim.

### Participants

YP in the UK, born with CL/P and aged 16 to 20 years, were eligible to participate. Invitations were disseminated on existing social media networks of the UK-based charity, the Cleft Lip and Palate Association (CLAPA). YP who expressed interest in taking part were sent an information sheet, consent form and for those aged 16 or 17, a covering letter for parents/carers was provided. These communications were facilitated by the CLAPA Children and Young People's Services Coordinator. Following the focus group each participant was sent a voucher as a thank you for attending online.

Ten YP aged 16 to 20 years were recruited. Across the participants, their cleft care had been received from 5 of the 11 managed UK Cleft Clinical Networks.^4,23^ Two participants were aged 16 to 17 years and eight aged 18 to 20 years. Nine were female and one was male (see Table 1).

**Table 1.** Participant Characteristics.

Age	Focus group	Sex	Cleft phenotype	Ethnicity
16	1	Female	BCLP	White
17	1	Male	CP	White
18	2	Female	UCLP	White
18	2	Female	UCLP	White
18	2	Female	UCLP	White
19	2	Female	CP	White
19	2	Female	BCLP	White
20	2	Female	CP	White
20	2	Female	CP	White
20	2	Female	CP	White

Abbreviations: CP, cleft palate only; UCLP, unilateral cleft lip and palate; BCLP, bilateral cleft lip and palate.

### Procedure

Two focus groups were conducted using the online platform, Zoom.^24^ Informed consent for the study was obtained in advance and repeated verbally at the beginning of the focus group. One group was held for participants aged 16 to 17 years and one for participants aged 18 to 20 years. The divide in age group was chosen to differentiate those who were likely to be still at school and those who had left and were experiencing different life events.

The zoom poll and chat functions were used to capture responses to questions asked during the focus groups. All responses were recorded, and field notes were taken by the research team.

Each focus group session took place over a 2-h period and were facilitated by authors JD, ED, and SH. A YP's coordinator from CLAPA was present to support the YP in Group 1, as they were under 18, but the coordinator did not participate in the discussions. At the end of each focus group, we asked all YP to contact us if there were any further questions or concerns and provided our email addresses for contact. The focus group recordings were transcribed verbatim.

### Nominal Group Technique

**Figure 1.**
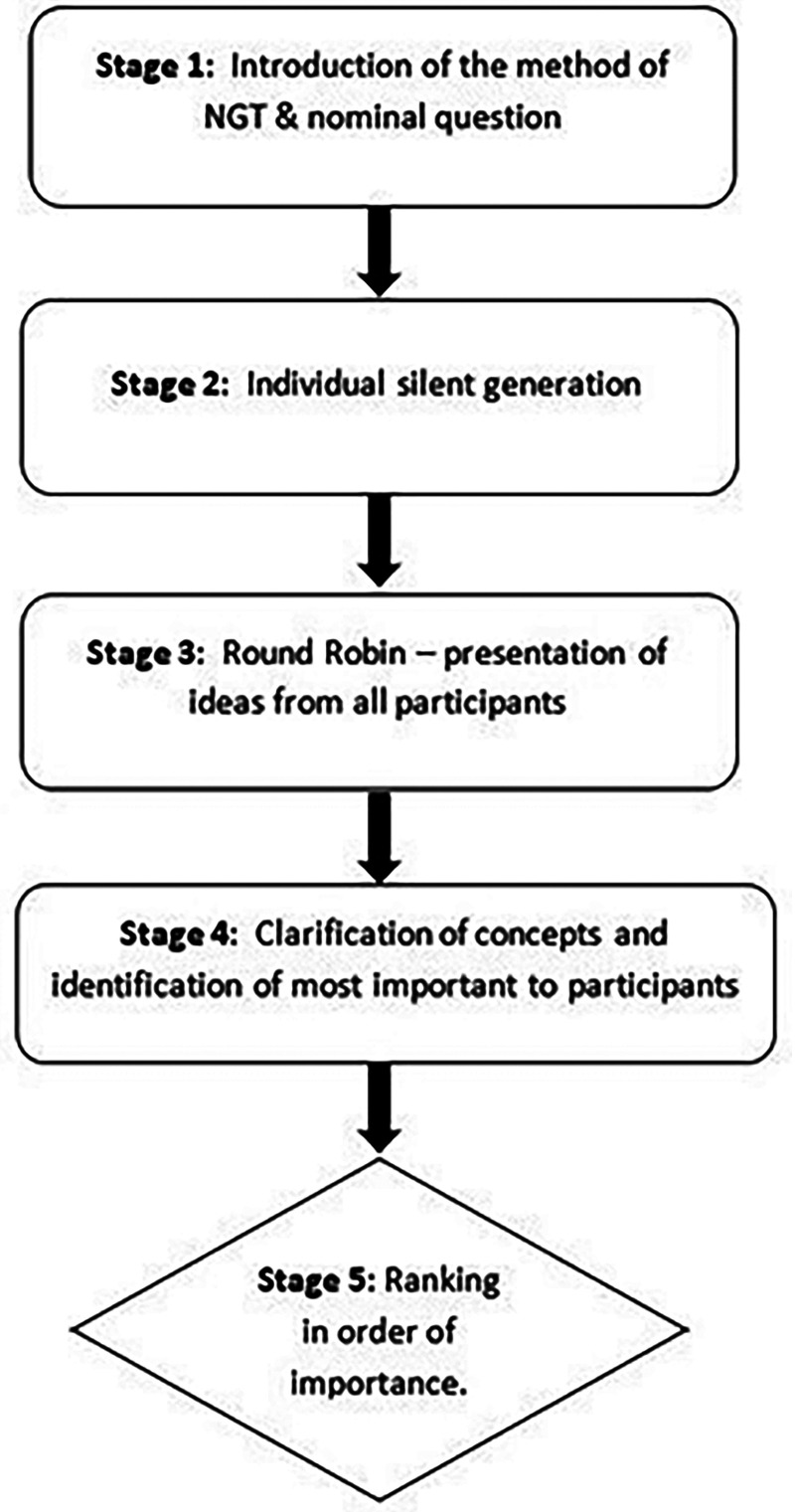
Summary of the 5 stages of nominal group technique (NGT).

NGT was used to facilitate the focus group discussions. The aim of this technique is to achieve group consensus and plan future actions on the topic under investigation.^25^ The process involves 5 steps of problem identification, solution generation, and decision-making^26^ (summarized in Figure 1).

The participants were asked to respond to questions presented by the facilitator, anonymously recording their answers on the Zoom poll. The questions asked were:

- 1. If you were invited to attend an appointment with the cleft team now, what would be the things that would be most important to you to be discussed/checked or measured?
- 2. What do you think are the greatest challenges experienced at this age, for YP born with CL/P?

Participants were then asked to group their responses to questions 1 and 2 into related concepts and subsequently rank these in order of importance. This approach has been shown to generate unique ideas, provide balanced participation between group members, increase feelings of accomplishment, and subsequently promote greater satisfaction with idea quality and group efficiency.^27^ It has been considered viable to conduct this method online.^28^

### Reflexive Thematic Analysis

The data collected during the 2 focus groups were analyzed using RTA to identify themes. RTA allows a critical realist epistemology to focus on the identification of broad themes whilst concentrating on the participants’ experience.^29^ Analysis was inductive and followed Braun & Clarke's (2022) 6-stage process. The first stage is to become fully acquainted with the data, the second stage is the coding of the data, the third stage is identification of themes, the fourth is the reviewing of themes, the fifth is the defining and naming of themes and the sixth stage is the final report of the findings.^30^

Analysis was led by JD and ED and discussed and refined with the rest of the authorship team.

### Reflexivity

The 2 lead authors and focus group facilitators both held roles within specialist cleft services; one is a Specialist Speech and Language Therapist JD and the second a Clinical Researcher with a surgical background ED. The third focus group facilitator and author is a Senior Research Fellow and Health Psychologist SH. All 3 facilitators were female, none had prior established relationships with any of the focus group participants. However, at the start of the groups, the facilitators introduced themselves with a brief outline of who they were professionally. Throughout the study, discussion and collaboration took place between the authors to reduce any impact these personal experiences may have had on the analysis.^31^

Ethical approval for the study was obtained from the Faculty of Health Science Research Ethics Committee (FREC), University of Bristol, reference number 9182.

## Results

### Responses to Question 1: What Should be Discussed at an Appointment?

After the introduction to the methodology (Figure 1), stage 2 and stage 3 of NGT generated 6 concepts from the YP aged 16 to 17 years in response to question 1 regarding the things that they considered would be most important to them to be discussed/checked or measured at an appointment, while the older age group generated 16 concepts (see Table 2).

**Table 2.** Generation of concepts—question 1.

YP aged 16 to 17	YP aged 18 to 20
Additional treatments Additional surgeries Difficulties experienced Any support available (eg, CLAPA) Appearance, school etc What do YOU (YP) want and why	Progress Mental Health Future treatments available Attitude toward future What YOU (YP) want Treatment/cosmetic surgery Clear plan of where treatment is going Appearance options Counsellor before operations Any issues YOU (YP) want to address Any questions YOU (YP) want answered Any other concerns Support mentally Access, how to get back in contact after discharge How to explain how you feel to your family Psychological stuff—post surgery/coping

Stage 4 of NGT, clarification of concepts, led to the creation of 2 core concepts from the age 16 to 17 group, which were felt to encompass the 6 silently generated concepts. These were:

- appearance/physical issues
- mental health.

The final stage (ranking) for this group resulted in both concepts being considered of equal importance.

In the older age group, clarification of concepts led to the identification of 3 core concepts which were felt to be of prime importance:

- re-accessing care.
- mental health.
- appearance/physical issues.

The final ranking stage for this group resulted in re-accessing care as the top-ranked concept and the remaining 2 being considered of equal rank.

### Responses to Question 2: What are the Greatest Challenges Experienced at This Age?

Stage 2 and stage 3 of NGT generated 7 concepts from the YP aged 16 to 17 years, while the older age group generated 15 concepts (see Table 3).

**Table 3.** Generation of Concepts—Question 2.

YP aged 16 to 17	YP aged 18 to 20
Appearance Confidence Wellbeing School Self-doubt Feeling happy with oneself Speech/hearing	Mental Health Self-confidence (new environments—Uni, jobs, etc) Self esteem Appearance—especially in the age of social media Physical differences Choice to change appearance or not Feeling isolated Worries of speech Worries of hearing Speech differences & language Not being able to relate Being in control over Cleft Care Knowing how to re-access Cleft Team Education (not wanting to miss time from education) Away from home—different local Cleft Team how to re-access care?

Stage 4 of NGT, clarification of concepts, led to the creation of 2 core concepts from the age 16 to 17 group, which were felt to encompass the 7 silently generated concepts. These were the same as for question 1:

- appearance/physical issues
- mental health.

The final ranking stage for this group once again resulted in both concepts being considered of equal importance.

In the older age group, clarification of concepts led to the identification of the same 3 core concepts as in question 1 which were felt to be of prime importance:

- re-accessing care
- mental health
- appearance/physical issues.

The final ranking stage for this group resulted again in re-accessing care being the top ranked concept and the remaining 2 being considered of equal rank.

**Figure 2.**
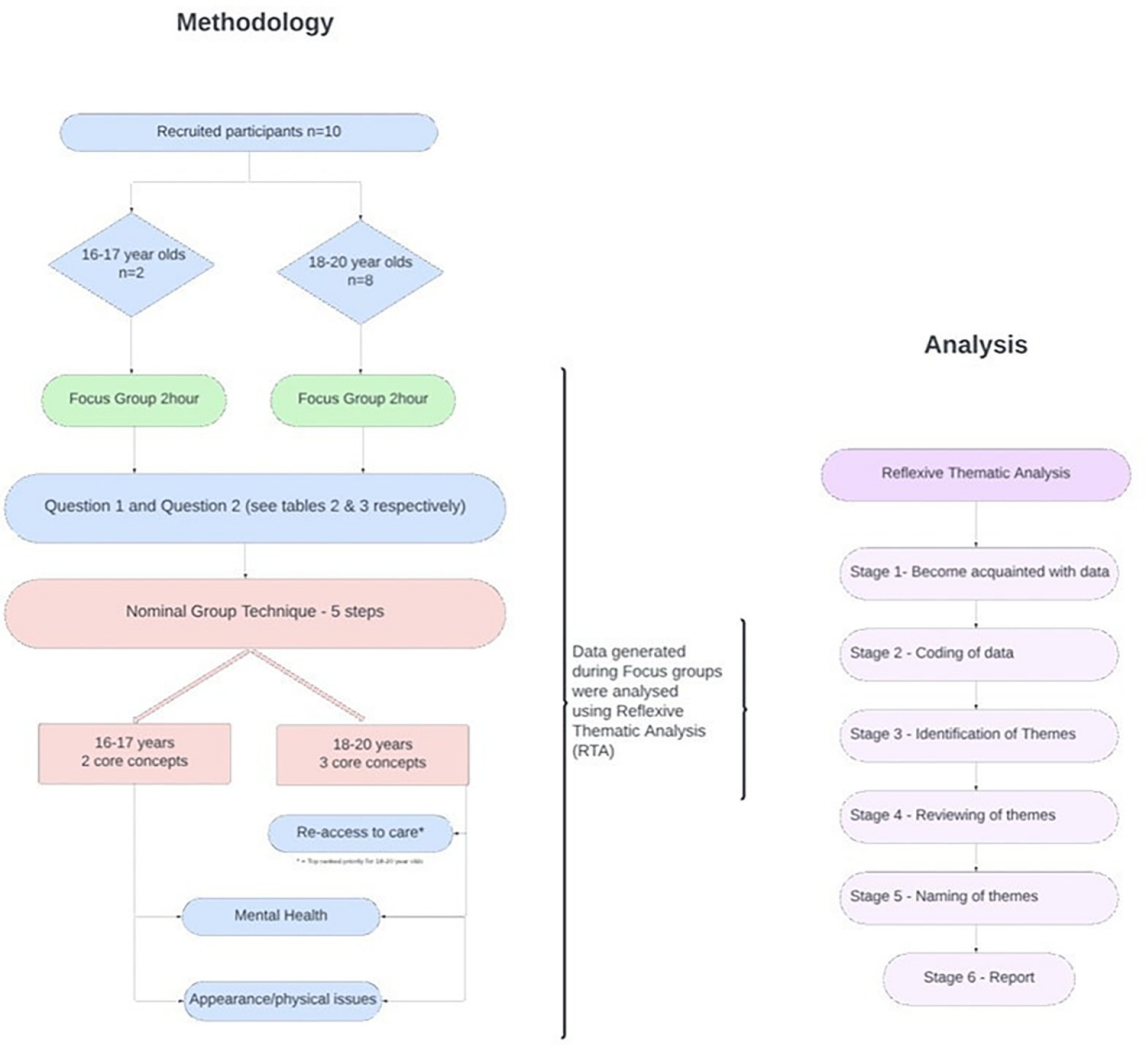
Methodology and analysis flowchart.

This process is summarized in the flowchart in Figure 2.

### Results of Reflexive Thematic Analysis

From the YP's narratives throughout both focus group sessions, 3 themes were identified that encapsulated the YP's views on approaching the end of routine care: Theme 1—Psychological well-being is not a constant, Theme 2—Asking and listening—the cleft clinic appointment, and Theme 3—Approaching end of routine care and re-accessing cleft services.

**Theme 1—Psychological well-being is not a constant**

During the NGT element of the group, all the YP agreed that the most important outcome was overall psychological well-being and how this was related to satisfaction with appearance/physical concerns and self-confidence. YP talked about how well-being fluctuates with age and different life experiences and therefore how it is important to be regularly monitored by cleft teams as they get older. They spoke of how their cleft related appearance/visible difference or awareness of their speech and hearing differences changes depending on their environment and that new, and often challenging environments could have a significant impact on their psychological wellbeing and self-confidence.

Like last year it wasn’t a concern for me, but this year, it is a concern for me.

All the personal struggles you go through, because of your appearance or because of your speech or your hearing, the worry and the anxiety that can bring on to your life, especially as you kind of move into adult life as well as all the other kind of external stressors of exams, and you know moving out and going to uni and getting a job and all that kind of stuff all happens at once, it's really good to know that your cleft team is there to support you.

As you grow older some things change, you might realize there's something that you want addressing which you didn't realize when you were younger.

Psychological well-being was also mentioned specifically in relation to the impact social media has on the YP with a visible difference. Using social media leads YP not only to critically scrutinize their appearance, like their peers, but also to contend with specific negative feedback relating to their visible difference, from those whom they share their image with on social media.

I feel like particularly you know when you have like a physical kind of difference in your appearance, no matter how large or small, like it's always the biggest thing that you notice in yourself so almost it's about not only kind of support in terms of what other people can scrutinise about you also what you can scrutinise about yourself.

Physical appearance is a lot more scrutinized over social media.

What you put on social media can be kind of really harshly criticized by people.

All the YP discussed how being members of support groups such as CLAPA helped their psychological wellbeing, as being able to share their experiences with other YP with cleft conditions provides important peer support to enhance their confidence.

I’ve been part of CLAPA since I was very little… it's given me a lot more confidence to talk so openly about things, meeting other people through it and also hearing about other people, and you know building that confidence as a group.

**Theme 2—“Asking and listening”—The Cleft Clinic appointment.**

YP reported that their experiences of cleft clinic appointments were important. They discussed the need to be provided with information, options and support when making decisions about potential further treatments, as this would enable them to make informed decisions about their treatment.

Being made aware what is available to you or having the opportunity to talk to your consultant about what you want, or what's the matter and then being able to give you like options.

As you grow up and you feel more informed… you feel I can, I want to make these decisions.

They felt this would also reduce the likelihood of clinicians viewing them as “a project,” with the sole focus of trying to make them look “normal.”

To ask you…if you want to change anything…surgeons sometimes look at you in you know you’re their projects… what can we do to make you perfect and sometimes I think they’re getting it a bit wrong.

YP raised the importance of being listened to by the cleft team so that they felt in control of their treatment.

The best thing for them to do is listen.

The way that clinicians asked how the YP were doing, was discussed, with all YP preferring a direct approach such as,

How are you, how's school, how are you finding things. You know, is there anything specific you're experiencing because you have a cleft.

The importance of the language used during the consultation was critical to ensure understanding for the YP. This was the case too for written documentation such as consent forms, so that the YP could fully understand and be in control of their treatment.

Consent forms they give you all the medical jargon…you’re like what's that even mean and they’re like it's fine just sign it and you’re like I don’t know what I’m signing.

YP said that sometimes it was difficult to talk to their family about the emotional impact that their condition and possible further treatments may have on their mental health. They said they would therefore like to have the choice of having their parents present or not when making decisions about further treatment.

As you get older you know you might not want your parents there, you might want to discuss things without sort of parents being there.

To help with decision making, YP said it was important to be able to access psychological support at this age as they were experiencing numerous challenges. Including having a visible difference, with their confidence/self-esteem, school, speech differences, hearing, and that they often needed help to make decisions about whether to change their appearance, or not.

All the personal struggles you go through, because of your appearance or because of your speech or your hearing, the worry and the anxiety that can bring on to your life, especially as you kind of move into adult life.

As well as being listened to during the clinic appointment, the YP discussed the importance of the team being aware of issues highlighted and raised during the focus group. Such issues were around potential flexibility when arranging appointments.

I don’t want my appointments to get in the way of school, exams, university.

The YP said they were happy to attend clinic appointments at this age and have outcomes measured.

And I feel like it'd be really good that you know that age range know that they can still access, you know actual treatment for the kind of consequences of having a cleft as well as whatever clinicians want to explore or measure around that point.

However, it is worth noting that this research did not have the opportunity to probe what the YP defined as “outcomes,” or what amount of time or effort they would be willing to expend on having measures (to define outcomes) taken.

It was important for the YP to know which clinicians would be present and why they need to be present. Specifically mentioned was the point of having students present and how this can stop the YP talking about sensitive issues. Ideas were suggested by the YP as to how to make them feel more at ease to talk openly.

Make sure the environment is comfortable for personal discussions to avoid being seen as an object, who could be modified.

I don’t see the point in all the students being there because then it's difficult to say I hate the way I look.

If you want to be able to help treat people, you need to help them feel comfortable to ask for that support or ask those questions.

As a young person having the confidence to be able to say to you know, a surgeon a consultant, you know, this is what I'm looking for.

I think honestly if a young person was to be put in front of a group of people and asked obviously quite sensitive questions, they might feel that they're quite put on the spot and you know, it just might be quite overwhelming.

The YP suggested that the decision of who should be present could be made via an App, text, or email prior to attending an appointment. They explained that previous experience of being asked to complete a questionnaire in the waiting room was felt to be too late. It made them feel uncomfortable and unable to speak up about their more sensitive concerns, in front of various cleft team members, observers and even their own parents.

You can say oh, please note we are expecting students to be attending this appointment if you’d like this not to go ahead, please email.

**Theme 3—Approaching end of routine care and re-accessing cleft services**

Specialist cleft teams in the UK provide clear guidance to patients at the point of discharge regarding how to reconnect with services but the data from our participants suggested that this information is not always fully understood or received. In particular the YP in the 18 to 20 year focus group wanted clear direction of how to re-access the team for information and support after discharge from routine appointments and when they no longer live at home.

I think you've gotta prioritise the access to care, cos if you can’t access anything you can't have the physical or the mental health support.

How I get back in touch after I’ve been discharged, that's where the bridge needs to be built.

I haven't had like my post op appointment, since I was 17 and I’m now 20.

I don't know what to do down here with how to get in contact with the team around here.

I was discharged when I was 15 so that's five and a bit years ago now, and I was effectively told then she said you don't need to worry about us she said, get back in touch, if you would like genetic testing when I wanted to have children, but that was kind of it, she didn't say get back in touch you know, to discuss other things.

The whole discussion through, that being able to access care is so important. Especially you know from the ages of 15 onwards, even into 20 s, 25 you know late late 20 s early 30 s even.

Surprisingly, they said they did not really understand each clinician's role and therefore did not know who the best person would be to try and contact for support when re-accessing the service.

The clinicians. I feel like if you…need to basically explain the roles…I feel like that would be a lot easier than just saying the clinicians.

One participant explained how they were unaware how to re-access the team for psychological support following discharge and didn’t know how they could get this support back,

I was discharged, and I was still kind of seeing a psychologist and I just got cut off.

In response to these issues around re-access they suggested that a “facilitator” as a point of contact within each cleft team and an annual brief text or email asking if they would like to contact their team, would be extremely helpful.

## Discussion

The aim of this research was to identify factors that YP born with CL/P, aged 16 to 20 years and living in the UK, believe are important and should be considered by cleft clinical teams. Focus group meetings were used to collect data using NGT and discussion. RTA was then used to analyze the qualitative data.

The results highlight that YP want to be seen by their cleft team up to and often beyond the age of 20 years. They suggest it is important to them to discuss treatments and raise questions about their future, and to discuss possible challenges they may be experiencing at an older age that they were not as a younger person. Perhaps it is not surprising that the top priorities ranked through NGT, to be discussed in clinic, mirrored the top challenges that the YP reportedly face at this age. The focus group discussions were further explored through RTA; 3 main themes were identified; Psychological well-being is not a constant; Asking and Listening—the cleft clinic appointment; Approaching end of routine care and re-accessing cleft services.

### Psychological Well-Being is Not a Constant

Psychological well-being can be considered a multilayered concept that incorporates a persons’ overall happiness, mental and emotional health and satisfaction with life.^32^

An improvement in well-being and acceptance of a cleft condition by the affected individual as they mature into adulthood has been previously reported in the literature.^33^ However, as previously found, well-being fluctuates in response to life events.^33^ This finding is supported by the YP in this study who discussed the need to be invited back to clinic in their late teens/early twenties to discuss concerns that may arise as they get older, and which could be addressed with specialist psychological support.

The YP also spoke about their well-being in relation to posting on social media. The need to be like their peers was important to them, but this could be difficult for individuals with a cleft condition who are living with a visible difference, as there was a fear of receiving negative comments. The sharing of images and the posting of “selfies” has become increasingly popular with teenagers and young adults.^34^ In a study of 790 adolescents aged 13 to 17 years from the University of Chicago, 94% of the YP reported using Instagram, Snapchat, and Facebook.^35^ There has been previous research to show that posting videos on social media can be empowering and promote acceptance^36^ of their cleft condition and so it may be suggested that the use of social media could be positive, with the right support networks in place, as it may help to normalize cleft conditions within the general population.

As well as psychological support from the cleft team, the importance of support from groups such as CLAPA was highlighted as extremely important in our NGT and focus group discussions. This has been discussed in previous literature with studies discussing the benefits of knowing others with cleft conditions^37^ and how support outside of the family is extremely beneficial.^38^ It is suggested that clinical teams regularly highlight to the YP the benefits of such support groups during their clinics.

### “Asking and Listening”—The Cleft Clinic Appointment

Being listened to and taking control of their treatment were highlighted by the YP as being important. When YP were listened to, they felt that clinicians were facilitating their active empowerment and enabling them to have control in their care.

Taking an interest in treatment was advised by adults with cleft conditions, in a previous study^39^ and has been found to support preparation and readiness for treatment and making decisions.^40^ Previous literature has reported that patients need to feel empowered to make decisions for themselves around treatment by being provided with appropriate information and that this is communicated in a way that they fully understand.^33^ In this study, YP raised concerns that they did not always understand words used or the discussion around treatment and this included the consent forms due to the medical jargon used.

Missing school and University at important stages of their lives needs to be taken into consideration. With the advent of virtual appointments now widely available since COVID-19, this model of service provision could continue and reduce the potential negative impact of both missed in-person appointments and absence from school, as well as being reliant on parents to transport and subsequently attend appointments with the YP.

Auditing outcomes for the purpose of evaluating and maintaining clinicians professional standards at the end of cleft care pathway, was discussed in this study. All YP were happy to have outcomes measured for this purpose, but with a request that this is discussed with them prior to the appointment so that they were fully aware of what to expect when they attend. The YP linked this with the presence of students and number of clinicians in the clinic room with some saying that they would be unlikely to talk about sensitive issues with many people being present.

### Approaching End of Routine Care and Re-Accessing Cleft Services

Re-access to cleft care as an older patient or following discharge from children services was raised as a significant concern by all the YP. The importance of having access to psychological support in particular was raised. Even though cleft teams in the UK routinely provide this information on discharge,^41^ participants in this research did not seem to be aware of how to reconnect with services. There was also confusion amongst participants regarding the roles of each member of the cleft team and this appeared to be a barrier to re-accessing further treatment. It was suggested by the YP that a named contact person within the team could act as a facilitator to enable reconnection with services when needed. This is supported by other research,^22^ which suggests a “named transition worker” within each cleft unit could act as a direct link between YP and the clinicians. An individual in the cleft team with whom the YP could build a relationship, in the same way that clinical nurse specialists within the cleft team already do with new parents at the start of the child's cleft care pathway.

## Limitations

Qualitative research typically involves small samples and the views of the participants in this research cannot be assumed to be generalizable to the wider population of YP born with CL/P. Participants who are recruited through organizations that offer support may be more accepting and open with their condition. Moreover, the lack of diversity amongst the participants means that it cannot be assumed that YP who differ from this sample in key characteristics will share their views. This has also been the case for previous studies which struggled to get a representative sample.^22^ In the time available to carry out this study, it was not possible to recruit a larger sample of 16- to 17-year olds and future research should consider creative ways to engage with this age group. The 18- to 20-year old focus group comprised an optimum number of participants^26^ but was still limited in its diversity.

Further research is needed to enable clear guidance to be developed which will address the points identified from the work described in this paper. Indeed, the Cleft@18-23 research program, funded by the UK National Institute of Health Research, has been set up to enable recruitment of a large sample of YP born with CL/P for mixed methods research. This work will also involve clinicians, facilitating co-design of recommendations and guidelines which are important to both the patient and clinical communities.

## Conclusion

The findings from this work suggest that YP in this study have ongoing needs throughout adolescence and into early adulthood and that they are finding it difficult to re-access support for these needs, even though guidance was generally provided. Whilst these findings provide support for the ongoing provision of services to this age group, at and beyond the end of routine care, there is a need for the issues identified in this work to be explored with a larger sample which reflects the diversity of the population of YP born with CL/P.

## Supplemental Material

sj-docx-1-cpc-10.1177_10556656241312494 - Supplemental material for Exploring the Needs of Young People Born With Cleft Lip and/or Palate Approaching End of Routine Care, in the UK Supplemental material, sj-docx-1-cpc-10.1177_10556656241312494 for Exploring the Needs of Young People Born With Cleft Lip and/or Palate Approaching End of Routine Care, in the UK by Julie Davies, Elaine Davidson, Sam Harding, Yvonne Wren and Lucy Southby in The Cleft Palate Craniofacial Journal

sj-pdf-2-cpc-10.1177_10556656241312494 - Supplemental material for Exploring the Needs of Young People Born With Cleft Lip and/or Palate Approaching End of Routine Care, in the UK Supplemental material, sj-pdf-2-cpc-10.1177_10556656241312494 for Exploring the Needs of Young People Born With Cleft Lip and/or Palate Approaching End of Routine Care, in the UK by Julie Davies, Elaine Davidson, Sam Harding, Yvonne Wren and Lucy Southby in The Cleft Palate Craniofacial Journal
