# Supplementary material for: Exploring the Needs of Young People Born With Cleft Lip and/or Palate Approaching End of Routine Care, in the UK
Source: Cleft Palate Craniofac J. 2025 Jan 17;63(4):654–63. doi: 10.1177/10556656241312494 (PMC12996362; doi:10.1177/10556656241312494)
Supplement: sj-docx-1-cpc-10.1177_10556656241312494 - Supplemental material for Exploring the Needs of Young People Born With Cleft Lip and/or Palate Approaching End of Routine Care, in the UK [file sj-docx-1-cpc-10.1177_10556656241312494.docx]

**Name(s) of Moderator____________________**

**Date_______________________________**

**Attendees___________________________**

**Appendix K**

**Young People’s Focus Group Topic Guide**

**Workshop Participants List**



**Introduction and Obtaining consent**

*Good afternoon/evening. My name is (XX), and these are my colleagues, (XY) and (XZ). We are Senior Research Associates from the University of .... (or other role as appropriate for other research team members who may be involved in running the group).*

*This research is led by ... from the University of ...*
*Thank you for attending our virtual focus group and for your time this evening.*

*You will all have received a consent form and participant information sheet via email prior to this meeting. The aim of this group is to obtain your views and opinions on the important outcomes to understand for young people aged 15-20 years who were born with cleft lip and palate. By outcomes we mean anything you feel is important to be discussed, measured or understood in relation to your health and wellbeing, as a result of being born with a cleft.*

*The cleft teams routinely check progress for children born with cleft lip and palate at age 5 years, however, later outcomes and ongoing needs, which reflect your longer-term experiences transitioning into adulthood are much less well understood.*

*Some of you may have been involved in the Cleft Care UK study which recruited children born with Unilateral Cleft Lip and Palate (UCLP) between 2005 - 2007 and then looked at outcomes across areas such as speech, hearing, dental health and overall wellbeing when they were aged 5. The aim of that work was to understand if outcomes for children born with a cleft had improved since cleft services were reviewed and changed in the early 2000s. Those who took part in CCUK are, like yourselves now reaching late adolescence/early adulthood. We would like to find out and understand how young people born with a cleft are doing at this older age and what support or treatment they might still want or need. In the future, with further funding we hope to better understand which treatments lead to better outcomes at this age, which ongoing services are needed, and which young people are most at risk of experiencing difficulties in young adult life and whether there are predictors from outcomes at age 5 that will inform future cleft care.*

*Before undertaking that work, it is important to determine what outcomes are appropriate and important to measure in this age group and this is the aim of the present study and these focus groups.*

*We are here to listen to your opinions on this. Your perceptions are what matter. There are no right or wrong, or desirable or undesirable answers. We would like you to feel comfortable saying what you really think and how you really feel, but there is no pressure at any time to have to speak up if you prefer not to. These discussions will help us to determine a core outcome set for this age group that will be the most meaningful and informative for clinicians and young people born with cleft.*

*Before giving your verbal consents this afternoon/evening, we would like to remind you that we will be recording the Zoom session and making notes of the discussion so that we do not miss anything you have to say. We will also be using the Zoom chat function to collect your opinions. Once we have completed the consent process, we will change your names on screen so that all comments and opinions shared in the chat function are anonymous.*

*The recordings will be transcribed by a member of the research team and made anonymous. The transcripts will be used to supplement notes taken during the meeting. To carry out the analysis of the focus group data, the transcript from the focus group will be shared with the other researchers in the team.*

*As you know the contents of what is discussed will be kept confidential. No one outside of this group will know who has said what. The group will last for two hours. There will be a comfort break half-way through.*

*For recording purposes, we would be grateful if everyone could keep their cameras on but mute themselves unless you wish to contribute in anyway. There is a lot we want to discuss, so at times we may need to move you along a bit.*

*So now to start, if everyone is happy with this, we need to ask you all to verbally consent to participation in this focus group. We would be grateful if each person could say their name and raise their hand, or say yes if they agree to each statement, we now read out from the consent form that was previously sent to you. Let us start from the top left of my screen…..*

*Thank you very much for consenting.*

**Introduction of focus groups attendees**

*Let’s begin by introducing ourselves and where abouts in the country you live.*

**Explanation of Nominal Group Technique**

*The method we are going to use for this group is called Nominal Group Technique. This is a method which involves a structured group session aiming to achieve group consensus and action planning on a chosen topic. We are using this technique because it has been shown to allow everyone to equally contribute.*

*The stages of the nominal group session are as follows:*

- *Stage 1: involves individual responses to questions – we will collect your responses via the Zoom chat function at this stage. This is referred to as a silent generation phase because it is really important for your responses to reflect your own opinions.*
- *Stage 2: involves clarification and consolidation – at this stage, we will ask you read out and clarify your responses one by one. We will collate these responses on a word document which we will share over the screen. As a group we will then merge similar/same items under one response.*
- *Stage 3: involves ranking the responses – Once we have collated the responses, we will ask you to rank your top 3 responses in order of importance in the zoom chat.*

*This process will be repeated for each of the main questions we have for you. Once we have completed this process for all the questions, we will have a short break, during which we will calculate the results from your ranked responses. We will then share these results with you after the break and have a short period of open discussion where you can share additional thoughts you have before finishing the session.*

*We will talk you through this as we go but as the process will be the same*  *for each question, it will become familiar as we go through.*

*Let’s begin, as you know, we would like to identify what you think is important to be discussed or measured at age 15 – 20 years.*

*As we mentioned earlier, for the purposes of confidentiality and anonymity we are going to rename you all now to the same name so that you can freely enter any thoughts or comments into the chat without identification.*

**Main body of discussion**

*We realise that some of you may have attended a clinic at this age and we look forward to discussing that with you in more detail later in this session but for now let’s move onto some questions about what you believe should be discussed and measured for young people such as yourselves aged 15 – 20 years*

***1) Do you think it is important that clinicians see you between the ages of 15-20 years of age?***

*(Zoom– poll yes/no to ease into discussion)*

*N of you said yes and N of you said no. We are going to go on and talk about what we might collect in a clinic for young people so before we start that, it would be good to understand why some of you said no – there is no pressure to give a response but would anyone who said no like to talk about why they said that?*

***2) If you were invited to attend an appointment at this particular age, could you name one thing that you feel would be most important to be discussed, checked or measured?***

*(Complete stages 1, 2, 3 of NGT or zoom chat / collate/rank)*

***3) What do you think are the greatest challenges experienced at this age, for young people born with a cleft?***

*Probe: speech, dentition, confidence…*

*(Complete stages 1, 2, 3 of NGT for each domain identified)*

**BREAK (Calculate rankings for the questions where NGT used above)**

***If we look at the top three responses to question 2 how do you think these are best assessed.***

***Probe: (****at what age, one to one, MDT, face-to-face, virtual...?)*

***(****discussion)*

***If we look at the top three responses to question 3 how can cleft teams support someone best in addressing the issues you have raised?***

***Probe: (****at what age, one to one, MDT, face-to-face, virtual...?)*

***(****discussion)*

If time permits, we will then move on to discuss the questions listed below:

***For those of you that have already attended a clinic at this age, what are your reflections of this appointment?***

***What obstacles do you think could prevent a young person attending a clinic at this age?***

***Which clinicians would you want to see in particular at this age? And do you think the clinic should be virtual or F2F?***

***How could the clinicians make you feel further at ease to discuss any issues that you may have at this age? Do you prefer seeing the clinician 1:1 or in a room with the rest of the team members? Would it be helpful to have a written summary at the end of the clinic so you can go back and read it after you’ve left the clinic?***

*(Probe: would completing a questionnaire before you see the clinician help you start a conversation with them?*

***Are there any services that you feel still need to be available to you after the age of 20 years?***

**Closure**

*Though there were many different opinions discussed regarding an adolescent research clinic it seems that... (e.g. most of you agree that …. But some of you think ... our job now is to collate all the findings from this focus group and the focus groups we are having with the various cleft clinicians.*

**Future**

*The information obtained from these focus groups will be used to determine a core outcome set for young people aged 15-20 years. This means a set of things that cleft teams could check or measure for all young people born with a cleft to understand how they are doing potentially across a number of areas and what else, if anything they need in terms of treatment or support. This information could then be used for future research to help inform and plan services that will maximise outcomes for children born with a cleft.*

*As a thank you for your time today contributing to the discussions you will receive a £40 voucher. This will be sent to you via email.*

*If you have any further questions or would like to find out any further information, please do not hesitate to ask as we will be more than happy to help. You can either contact myself or XY in the first instance and we can then pass on any comments or queries to ... who is leading this research. Our contact details are at the bottom of the Participant Information sheet that was emailed to you.*

*Before we finish is there anything that anyone would like to add, mention, or discuss?*

*Thank you very much for coming this afternoon/evening. Your time is very much appreciated, and your comments have been extremely helpful.*
